# Neural Progenitor Cell- and Developing Neuron-Derived Extracellular Vesicles Differentially Modulate Microglial Activation

**DOI:** 10.3390/ijms26157099

**Published:** 2025-07-23

**Authors:** Tsung-Lang Chiu, Hsin-Yi Huang, Hock-Kean Liew, Hui-Fen Chang, Hsin-Rong Wu, Mei-Jen Wang

**Affiliations:** 1 Division of Neurosurgery, Neuro-Medical Scientific Center, Hualien Tzu Chi Hospital, Buddhist Tzu Chi Medical Foundation, Hualien 970, Taiwan; poluschiou@gmail.com; 2 Department of Medical Research, Hualien Tzu Chi Hospital, Buddhist Tzu Chi Medical Foundation, Hualien 970, Taiwan; hyhuang@tzuchi.com.tw (H.-Y.H.); hockkean@hotmail.com (H.-K.L.); check172@tzuchi.com.tw (H.-F.C.); rong3016@gmail.com (H.-R.W.)

**Keywords:** microglia, neural progenitor cells, developing neurons, extracellular vesicles, Toll-like receptor 7, MAPKs, NF-κB

## Abstract

The developmental processes of microglia follow a general pattern, from immature amoeboid (activated) cells to fully ramified (inactivated) surveilling microglia. However, little is known about the mechanisms controlling the transition of microglia from an activated to an inactivated state during brain development. Due to the complexity of microenvironmentally dynamic changes during neuronal differentiation, interactions between developing nerve cells and microglia might be involved in this process. Extracellular vesicles (EVs) are cell-released particles that serve as mediators of cellular crosstalk and regulation. Using neural progenitor cells (NPCs) and a long-term neuron culture system, we found that EVs derived from NPCs or developing neurons possessed differential capacity on the induction of microglial activation. The exposure of microglia to NPC- or immature neuron (DIV7)-derived EVs resulted in the higher expression of protein and mRNA of multiple inflammatory cytokines (e.g., TNF-α, IL-1β, and IL-6), when compared with mature neuron-derived EVs. Exploration of the intracellular signaling pathways revealed that MAPK signaling, IκBα phosphorylation/degradation, and NF-κB p65 nuclear translocation were strongly induced in microglia treated with NPC- or immature neuron-derived EVs. Using a pharmacological approach, we further demonstrate that Toll-like receptor (TLR) 7-mediated activation of NF-κB and MAPK signaling cascades contribute to EV-elicited microglial activation. Additionally, the application of conditioned media derived from microglia treated with NPC- or immature neuron-derived EVs is found to promote the survival of late-developing dopaminergic neurons. Thus, our results highlight a novel mechanism used by NPCs and developing neurons to modulate the developmental phases and functions of microglia through EV secretion.

## 1. Introduction

Microglia are resident immunocompetent cells in the central nervous system (CNS). They play indispensable roles in brain development and the maintenance of homeostasis [1,2,3,4], while also potentially contributing to the development of brain diseases [5]. Microglia originate from primitive erythromyeloid progenitors in the yolk sac, beginning at embryonic day (E) 8.5 in the developing mouse brain [6,7,8,9,10]. These progenitors migrate into the brain at E9.5, and thereafter expand their population locally and distribute spatiotemporally in the CNS [11,12,13,14,15]. During early brain development, microglia have an amoeboid rather than a ramified morphology. This morphology has been reported to promote their flexible, locomotive migration and phagocytosis. As development progresses, microglia transform into ramified shapes acquiring long processes, which are commonly observed in the adult brain [3,12,13,16]. Microglia participate in important developmental functions including neurogenesis, neuronal survival and death, synapse maturation and elimination, axon fasciculation, and oligodendrogenesis [2,3,15,17]. Microglia are dynamic and present distinct morphology during different phases of development. Once the blood–brain barrier is formed, which occurs early in development, the developmental effects of microglia can be solely attributed to intrinsic cues in the CNS. This developmental program can be controlled by both intrinsic genetic programs and extrinsic signals from their niche in the brain [3,16,18,19,20].

A growing body of evidence supports the idea that neurons inform microglia about their status and are capable of controlling microglial functions in the adult CNS. This neuron-to-microglia crosstalk occurs either through direct cellular contact or through the release of soluble molecules by neurons [21,22,23]. Extracellular vesicles (EVs) have recently been found to participate in cellular communication in the brain [24,25,26]. EVs, including exosomes and microvesicles with a diameter of 30–150 nm and 100–1000 nm, respectively, are crucial carriers that mediate intercellular signaling [27]. Their cargoes include proteins, lipids, mRNAs, miRNAs, and non-coding RNAs, which can be transferred from source cells to target cells [28,29]. Within the nervous system, neurons and glia (e.g., astrocytes, oligodendrocytes, and microglia) have been observed to release EVs that could target certain cell types [30,31,32]. An increasing number of studies have shown that neuron-derived EVs play a vital role in neuron–microglia interactions [33,34,35,36]; for example, neuronal exosome-delivered miRNAs, depending on the source neurons, can either suppress or facilitate the proinflammatory activation of microglia [34,36].

In the rodent developing brain, an amoeboid (activation)-to-ramified (inactivation) morphological transformation has been observed in microglia [3,15,16]. To tightly control these developmental processes, the brain may provide an activated and restricted micromilieu during embryogenesis. In contrast to the more detailed knowledge about how microglia modulate neuronal development [2,3,37], the regulatory effects of developing nerve cells on microglial activity remain elusive. EVs are found throughout the developing nervous system [38,39]. It has been shown that neural stem/progenitor cells (NPCs) can liberate EVs [40,41,42], thus representing a possible mode of communication between NPCs or differentiating neurons and microglia, mediated via EVs during brain development. In the current study, we investigate whether NPC- and developing neuron-derived EVs might differentially modulate microglial activation and aim to elucidate the possible mechanisms of this modulatory effect.

## 2. Results

### 2.1. Characterization of Developing Neurons and Isolated EVs

**Figure 1:**
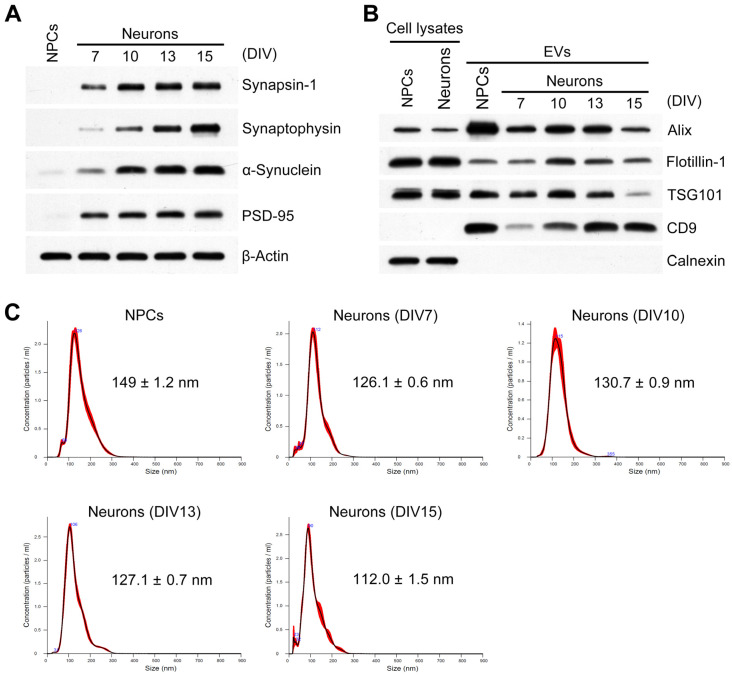
Characterization of developing neurons and EVs. (**A**) Molecular maturation of mesencephalic neurons in culture. Mesencephalic neurons were cultured for the indicated times. Cells were lysed and equal amounts of proteins were subjected to Western blotting. Specific antibodies for the different synaptic proteins were used, with β-actin as the loading control. (**B**) Western blot analysis of EV and non-EV marker proteins, including Alix, TSG101, flotillin-1, CD9, and calnexin (ER marker) in the cell lysates and EVs. (**C**) Size distribution of EVs derived from NPCs and developing neurons evaluated by nanoparticle tracking analysis. Estimated mean particle sizes are represented in the profiles.

For the isolation of EVs secreted from developing neurons, a long-term cell culture system described previously in research on neuronal maturation [43,44] was used. We determined the mature status of the long-term cultured mesencephalic neurons according to the expression of synaptic proteins. As shown in Figure 1A, the levels of mature neuron-expressed pre- or post-synaptic proteins increased in a time-dependent manner in cultured neurons, whereas these synaptic proteins were not detected in NPC cultures. These results indicate that the long-term culture of neurons can be used as a model for neuronal development. EVs were isolated from the culture medium of NPCs or neurons via ultracentrifugation. Western blotting and nanoparticle tracking analysis (NTA) were carried out to verify the EVs [27]. The Western blot analyses showed that the EV-specific proteins Alix, Flotillin-1, Tsg101, and CD9 were present in the EV preparations derived from NPCs and neurons (Figure 1B). In contrast, calnexin, a marker protein for the endoplasmic reticulum, was not found. In addition, NTA revealed that the mean particle size of NPC- and developing neuron-derived EVs was within the range of 110~150 nm (Figure 1C). Thus, the EVs were isolated successfully.

### 2.2. Differential Regulation of Microglial Activation by NPC- and Neuron-Derived EVs

**Figure 2:**
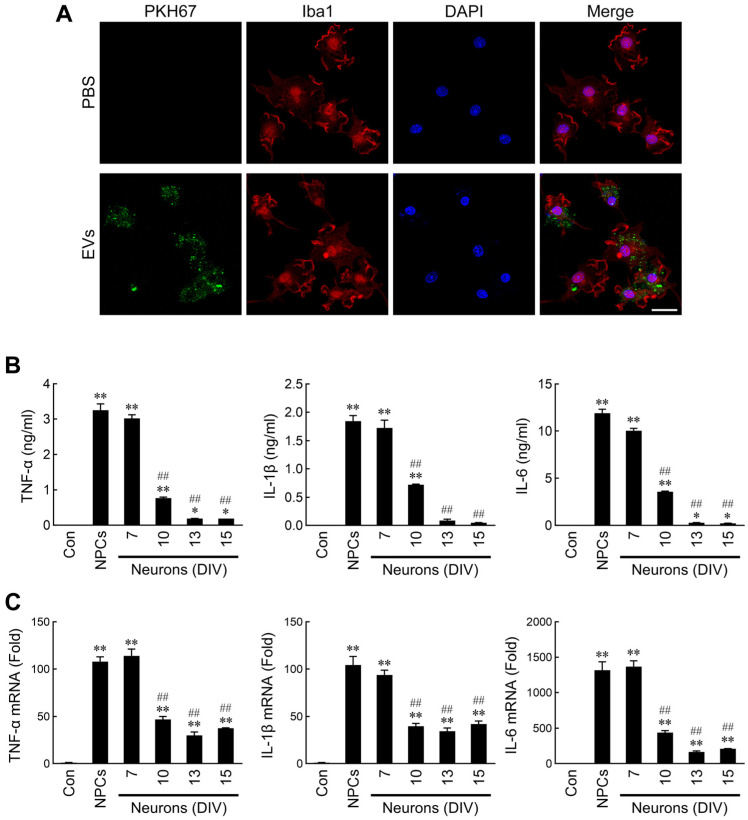
Effects of NPC- and developing neuron-derived EVs on microglial activation. (**A**) EVs were labeled with the dye PKH67 (green) and then added to microglia. After 1 h of incubation, cells were fixed, stained with Iba1 antibody (red) and DAPI (blue), and analyzed by confocal microscopy. Scale bar = 20 μm. Microglia were treated with PBS or EVs secreted from NPCs or mesencephalic neurons of different culture age for 4 h (**C**), 10 h ((**B**), TNF-α), or 24 h ((**B**), IL-1β and IL-6). The released TNF-α, IL-1β, and IL-6 levels (**B**) were measured by ELISA. Expression of cytokine mRNA (**C**) was quantified by real-time RT-PCR. Data are presented as mean ± SEM for three independent experiments. Data were analyzed by one-way ANOVA followed by Scheffe’s post hoc test. * *p* < 0.05; ** *p* < 0.01 compared with control. ## *p* < 0.01 compared with NPC EVs.

The well-known ramified (resting state) and amoeboid (activated state) morphotypes of microglia have been observed in the developing brain. It is well-known that the functions, activation status, and morphology of microglia are closely related [45,46]. To date, most studies have considered the release of proinflammatory cytokines as an index of microglial activation. To determine whether NPC- or neuron-derived EVs can regulate microglial activation, cytokines released into microglia culture supernatants following treatment with EVs were assessed. First, we labeled the EVs with PKH67 dye to evaluate whether EVs were taken up by microglia. As shown in Figure 2A, the labeled EVs were uptaken into microglia. Furthermore, the results indicated that dramatic production of TNF-α, IL-1β, and IL-6 was induced by NPC- and immature neuron (DIV7)-derived EVs, but not EVs from mature neurons (Figure 2B). To investigate whether the discrepant production of cytokines in microglia following the EV treatments was due to differential modulation of their mRNA expressions, real-time RT-PCR was performed to assess the mRNA levels. Consistent with the previous results, NPC- and immature neuron-derived EVs indeed induced higher levels of cytokine mRNAs (Figure 2C).

### 2.3. Divergent Activation of MAPK Signaling by NPC- and Neuron-Derived EVs

**Figure 3:**
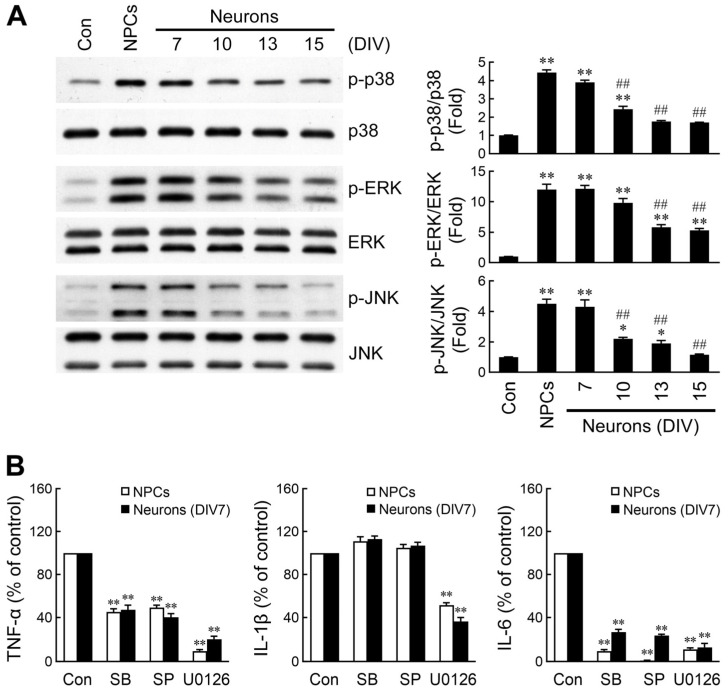
NPC- and developing neuron-derived EVs differentially induce activation of MAPK signaling in microglia. (**A**) Microglia were treated with PBS or EVs derived from NPCs or mesencephalic neurons for 1 h. Whole-cell extracts were prepared. Western analysis was used to determine EV-induced p38, ERK, and JNK phosphorylation. Data are presented as mean ± SEM for three independent experiments. Data were analyzed by one-way ANOVA followed by Scheffe’s post hoc test. * *p* < 0.05; ** *p* < 0.01 compared with control. ## *p* < 0.01 compared with NPC EVs. (**B**) Microglia were pretreated with SB203580 (SB, 10 μM), SP600125 (SP, 10 μM), or U0126 (10 μM) for 1 h, followed by exposure to NPC or immature neuron (DIV7) EVs for another 10 h (TNF-α) or 24 h (IL-1β and IL-6). Released cytokines were measured by ELISA. Data are presented as mean ± SEM for three independent experiments. Data were analyzed by one-way ANOVA followed by Scheffe’s post hoc test. ** *p* < 0.01 compared with respective control.

MAP kinase signaling cascades, including p38, ERK, and JNK, are well-known to be responsible for microglial activation [47,48]. To investigate whether these kinases are differentially activated by NPC- and neuron-derived EVs, microglia were treated with EVs for 1 h. Phosphorylation of the three MAPKs was analyzed by Western blotting. As shown in Figure 3A, EVs released from NPCs or immature neurons markedly induced p38, ERK, and JNK activation, whereas neuron-derived EVs elicited reduced phosphorylation of these MAPKs with increasing neuron culture age. Consequent to the above observations, the essentiality of these pathways for EVs-induced inflammatory cytokine production was determined. Microglia were incubated with SB203580 (a p38 inhibitor), SP600125 (a JNK inhibitor), and U0126 (an ERK inhibitor) prior to stimulation with NPC- or immature neuron-derived EVs. The production of TNF-α and IL-6 presented pronounced decreases in the presence of these inhibitors, whereas the release of IL-1β was attenuated after treatment with the ERK inhibitor (Figure 3B). Taken together, these results suggest that the stronger microglial activation mediated by NPC- and immature neuron-derived EVs can be attributed to the augmentation of MAPK signaling.

### 2.4. Discrepant Activation of NF-κB Signaling by NPC- and Neuron-Derived EVs

**Figure 4:**
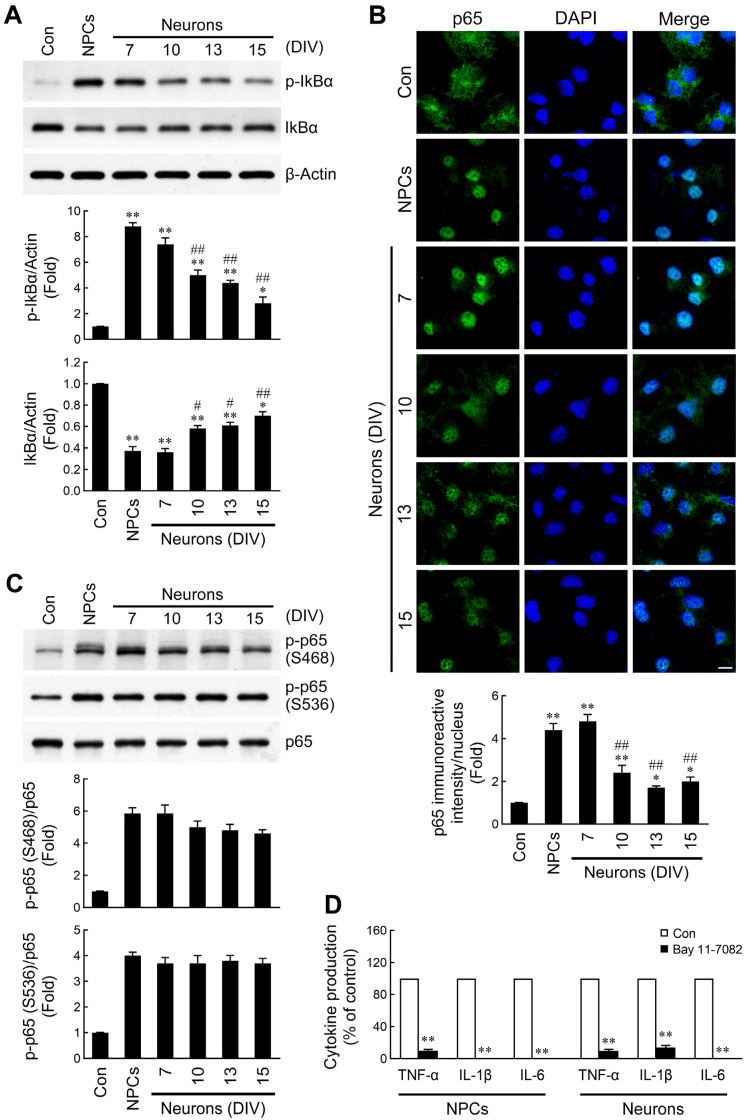
Discrepant activation of NF-κB signaling pathway following EV treatments. Microglia were treated with PBS or EVs derived from NPCs or mesencephalic neurons for 1 h (**A**,**C**) or 2 h (**B**). Whole-cell lysates were subjected to Western blotting using antibodies specific for total/phosphorylated (Ser 32) IκBα (**A**) or total/phosphorylated (Ser 468 and 536) NF-κB p65 (**C**). (**B**) After EV treatments, microglia were fixed and immunostained using anti-p65 antibody followed by counterstaining with DAPI. Representative confocal images are shown. The p65 fluorescence intensities in the nucleus were determined by image analysis. Scale bar = 10 μm. Data are presented as mean ± SEM for three independent experiments. Data were analyzed by one-way ANOVA followed by Scheffe’s post hoc test. * *p* < 0.05; ** *p* < 0.01 compared with control. # *p* < 0.05; ## *p* < 0.01 compared with NPC EVs. (**D**) Microglia were pretreated with BAY 11-7082 (an IκBα phosphorylation inhibitor, 2 μM) for 1 h prior to stimulation with NPC- or immature neuron-derived EVs for another 10 h (TNF-α) or 24 h (IL-1β and IL-6). Released cytokines were measured by ELISA. Data are presented as mean ± SEM for three independent experiments. Data were analyzed by paired *t*-test. ** *p* < 0.01 compared with control.

The NF-κB signaling axis has been demonstrated to be a critical determinant for the expression of many inflammatory genes [49,50]. Under resting conditions, NF-κB is sequestered in the cytoplasm through binding to a family of inhibitory proteins termed inhibitor κBs (IκBs). After phosphorylation of IκBα by the IκB kinase (IKK) complex, IκBα is degraded and releases NF-κB [51]. Therefore, we determined the effects of the EVs on IκBα phosphorylation and degradation. Similar to the above observations, the induction of IκBα phosphorylation and degradation in microglia treated with NPC- or immature neuron-derived EVs was stronger than that in the mature neuron-derived EV-treated groups (Figure 4A). Translocation of the transcription factor NF-κB into the nucleus is a downstream signal of IKK activation and IκBα degradation. Next, we also addressed nuclear accumulation of NF-κB p65 in microglia following treatment with EVs. After incubation with EVs, microglial cells were fixed and p65 immunofluorescence staining was performed. As shown in Figure 4B, remarkable fluorescence appeared in the nucleus, as observed in both NPC- and immature neuron-derived EV-treated microglia. However, the nuclear fluorescence signals were weaker in microglia incubated with mature neuron-derived EVs. These results indicate that the induction of nuclear translocation of NF-κB p65 is more pronounced by NPC- and immature neuron-derived EVs, when compared to mature neuron EVs. Post-translational modifications of NF-κB have been reported to be required for the complete activation of NF-κB-dependent gene expression [49,50]. Phosphorylation of the p65 subunit at specific amino acid residues is indispensable for the transactivation of target genes. Therefore, we included an assessment of p65 phosphorylation in our analysis. Microglia treated with EVs derived from NPCs or neurons showed an obvious induction of p65 phosphorylation at Ser468 and 536 (Figure 4C). However, there was no significant discrepancy in induction of p65 phosphorylation between NPC- and developing neuron-derived EVs.

To validate whether NF-κB signaling is involved in the upregulation of inflammatory cytokine production, microglia were pretreated with a NF-κB inhibitor (Bay 11-7082) followed by administration of EVs. The results demonstrated that pretreatment of cells with Bay 11-7082 abrogated EV-induced TNF-α, IL-1β, and IL-6 production (Figure 4D), indicating that the powerful capability of NPC- and immature neuron-derived EVs in terms of induction of cytokine expression was mediated by robust activation of the NF-κB signaling pathway.

### 2.5. NPC- and Immature Neuron-Derived EVs Activate Microglia via Toll-like Receptor 7 (TLR7) Signaling Axis

**Figure 5:**
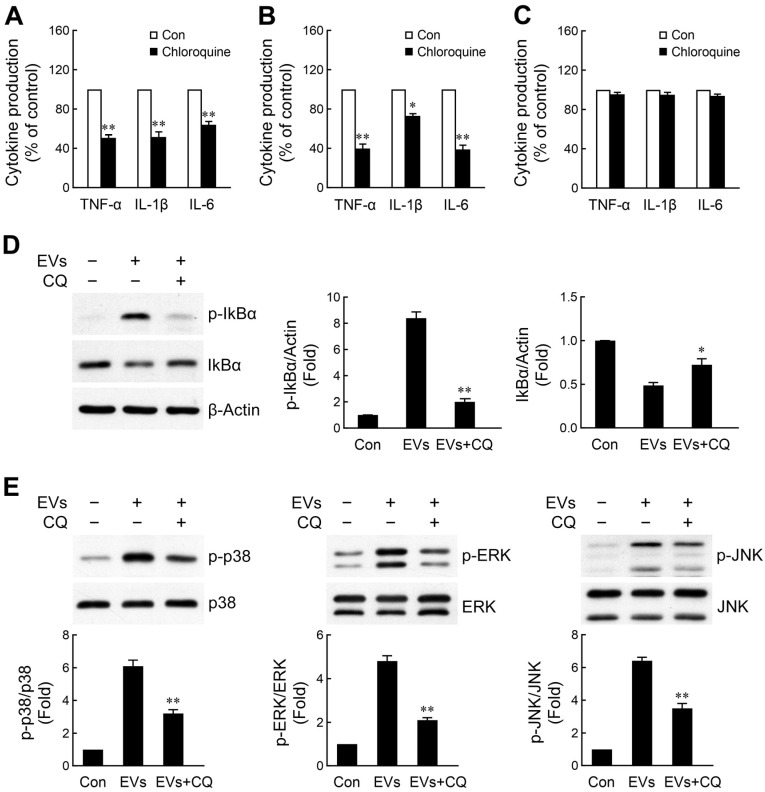
EV-mediated microglial activation involves TLR7-dependent signaling pathway. Microglia were preincubated with the TLR7 inhibitor chloroquine (10 μM) for 1 h before stimulation with NPC- (**A**) or immature neuron (**B**) -derived EVs or LPS (10 ng/mL) (**C**) for another 10 h (TNF-α) or 24 h (IL-1β and IL-6). Released cytokines were measured by ELISA. Data are presented as mean ± SEM for three independent experiments. Data were analyzed by paired *t*-test. * *p* < 0.05; ** *p* < 0.01 compared with control. (**D**,**E**) Microglia were pretreated with 10 μM chloroquine (CQ) for 1 h, then stimulated with NPC-derived EVs for 1 h. The levels of phosphorylated and total IκBα (**D**), p38, ERK, and JNK (**E**) proteins were detected by Western blotting. Data are presented as mean ± SEM for three independent experiments. Data were analyzed by paired *t*-test. * *p* < 0.05; ** *p* < 0.01 compared with EVs alone.

It has been suggested that EV cargo miRNAs play a critical role in the cellular crosstalk. Single-stranded RNAs, including miRNAs, are known to activate the endosomal receptors TLR7 and TLR8 in a sequence-specific manner [52,53,54]. Some miRNAs have been recognized as regulators in the EV-mediated activation of macrophages and microglia via binding to TLR7 [55,56,57]. To examine whether TLR7 was responsible for engaging NPC- or immature neuron-derived EVs, thus resulting in inflammatory cytokine production, microglia were pretreated with chloroquine (a TLR7 inhibitor) [55,57] followed by exposure to both EVs. Chloroquine inhibits TLR7 signaling through two primary mechanisms. First, as a weak base, chloroquine accumulates in endosomes and lysosomes, where it increases the intravesicular pH. This prevents the acid-dependent proteolytic processing and activation of endosomal TLRs [58]. Second, chloroquine can directly bind to nucleic acids, masking them from recognition by TLRs. This physical interference prevents TLR activation even in the presence of ligands, independent of pH modulation [59]. As shown in Figure 5A,B, pretreatment of cells with chloroquine significantly inhibited the stimulation of cytokine secretion by both EVs. As expected, chloroquine failed to abrogate LPS, a TLR4 ligand, -induced the expression of inflammatory cytokines (Figure 5C). The EV-induced production of inflammatory cytokines might be mediated through intracellular phosphorylation signaling cascades activated by TLR7, including NF-κB and MAPK signaling pathways [60,61]. Therefore, we next sought to determine the effect of blockade of this receptor on EV-activated NF-κB signaling. Analysis of key proteins in the NF-κB signal transduction cascade by Western blotting revealed that EV-induced IκBα phosphorylation and degradation was obviously abated by chloroquine (Figure 5D). Furthermore, chloroquine was able to block EV-induced phosphorylation of the three considered MAPKs in microglia (Figure 5E). Collectively, our findings indicate that EV-induced TLR7-mediated activation of the NF-κB and MAPKs signaling pathways may contribute to microglial activation.

### 2.6. NPC- and Immature Neuron-Derived EV-Activated Microglia Promote Developing Dopaminergic (DA) Neuron Survival

**Figure 6:**
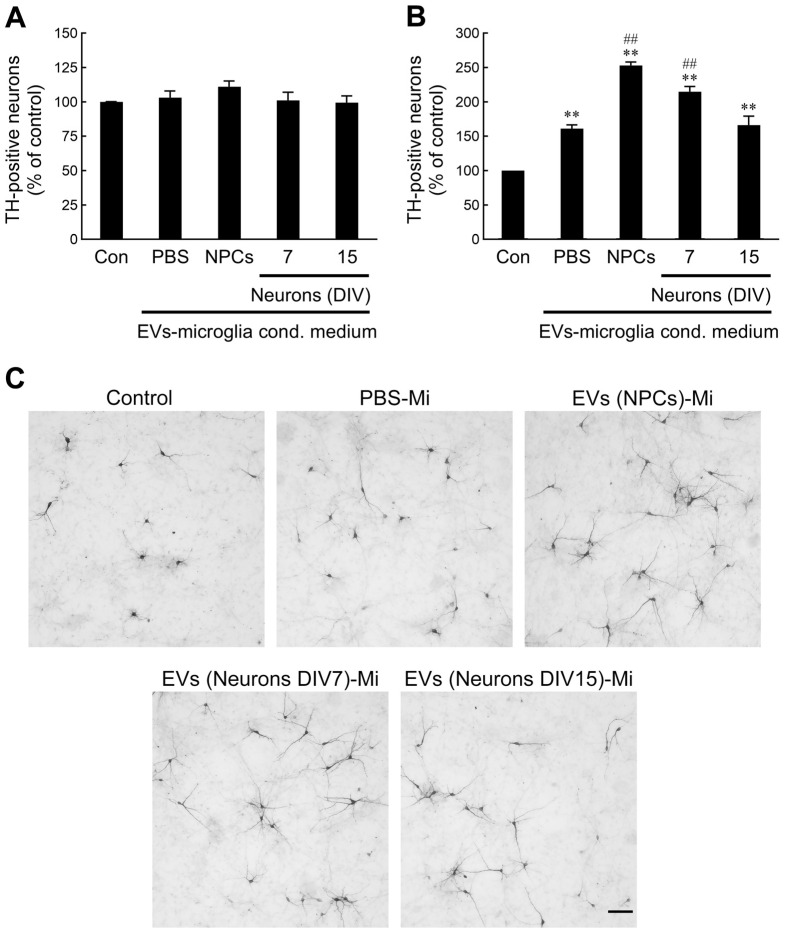
NPC- and immature neuron-derived EV-activated microglia promote the survival of differentiating dopaminergic neurons. Ventral midbrain precursor cultures were treated with unconditioned medium (control) or conditioned medium (35%) from PBS or EV-stimulated microglia for 3 days (**A**) or 6 days (**B**). TH immunocytochemical analysis was carried out. Neurons immunostained with TH were counted in the entire surface area of each culture well (i.e., 1.9 cm^2^). Data are presented as mean ± SEM for three independent experiments. Data were analyzed by one-way ANOVA followed by Scheffe’s post hoc test. ** *p* < 0.01 compared with unconditioned medium. ## *p* < 0.01 compared with PBS-treated conditioned medium. (**C**) TH immunostaining shows an increase in the number of TH-immunoreactive cells at 6 days after treatment with conditioned medium from NPC- or immature neuron-derived EV-stimulated microglia. Scale bar = 100 μm.

In the developing brain, the presence of a bidirectional crosstalk between developing neurons and microglia may serve as a modulating factor affecting proper neural development. We hypothesized that the release of factors from microglia following treatment with EVs may exert a feedback response to neural precursors. To test this hypothesis, conditioned media from non-treated or EV-treated microglia were introduced into precursor cultures derived from the ventral mesencephalon (VM). Treatment of precursor cells with unconditioned or EV-treated microglia-conditioned media led to no significant difference in the number of TH^+^ cells at 3 days in vitro (Figure 6A), indicating that conditioned media from microglia treated with NPC- or neuron-derived EVs did not result in differential induction of the TH-positive phenotype. In our previous study, it was demonstrated that differentiating DA neurons started to undergo spontaneous cell death after 4 days in vitro when cultured in differentiation medium [62]. To determine the effect of conditioned medium obtained from EV-treated microglia on DA neuron survival, VM precursor cultures were incubated with conditioned media for 6 days. We found that spontaneous DA neuronal death was markedly suppressed in response to conditioned medium from PBS-treated microglia relative to unconditioned medium (Figure 6B). Notably, the survival of DA neurons was further increased following exposure to conditioned media from NPC- or immature neuron-derived EV-treated microglia. Additionally, the surviving DA neurons were observed to have longer and healthier neurites compared to those in the control conditioned medium (Figure 6C). Our data suggest that NPC- or immature neuron-derived EVs induce microglial activation, which might in turn improve the survival of developing DA neurons.

## 3. Discussion

At different stages of development, microglia exhibit different morphological phenotypes. This variety of phenotypes allows for a variety of functions in the developing CNS. The maturation of microglia during development follows a general pattern from immature amoeboid (activation) or poorly ramified cells to fully ramified surveilling microglia (inactivation) [2,3]. Increasing evidence has demonstrated that microglia acquire a specialized morphology that is tailored to changes in the developing brain through a combination of gene regulation and responses to local cues. Distinct transcription factors have been found to participate in regulating the developmental phases of microglia [18,20,63]. Zusso and colleagues found that the Runt-related transcription factor 1 (Runx1) suppresses amoeboid microglia proliferation and guides these cells toward a ramified inactivated phenotype in the postnatal mouse forebrain [63]. Furthermore, a recent work has reported that the zinc finger transcription factor SALL1 is indispensable for the conversion of early microglia (amoeboid morphology) toward a more mature status (ramified morphology) during development [20]. Additionally, cell culture studies have revealed that astrocytes facilitate microglial ramification via the release of purines and cytokines [64,65]. McKinsey et al. [66] demonstrated that radial glia-expressed integrin α_v_β8 drives microglial maturation through the upregulation of microglia-expressed TGFβ1. Taken together, these findings suggest that the developmental phases of microglia can be modulated by both intrinsic factors and communication with surrounding cells.

Given that microgliogenesis and neurogenesis occur concomitantly in the developing brain, it is expected that neuronal progenitors or developing neurons and microglia constantly interact with each other and modify each other’s developmental programs. Previous studies have demonstrated the role of endogenous NPCs in regulating the fates of microglia (e.g., proliferation, migration, phagocytosis, and activation) through the ablation of NPCs in vivo or addition of NPCs in vitro [67,68,69,70]. Most of these effects of NPCs on modulating microglia functions are mediated by the secretion of soluble ligands [70]. EVs secreted by cells that may contribute to cell–cell crosstalk through the transmission of proteins, nucleic acids, and bioactive lipids [28,29]. Emerging evidence has highlighted the important roles of EVs in intercellular communication between neurons and microglia. Morton and colleagues [40] revealed that neonatal subventricular zone (SVZ) NSCs release EVs in vitro and in vivo. The incubation of microglia with NSC EVs was shown to augment the release of proinflammatory cytokines, and EV uptake resulted in a shift in the microglial morphology to a reduced number of cellular processes and a rounded appearance within the SVZ [40]. Consequently, NSC EVs may function as a non-canonical morphogen to control microglia. In the current study, we further demonstrated that EVs secreted by primary VM NPCs, immature neurons, and mature neurons exhibited discrepant effects on microglial activation. Notably, NPC- and immature neuron-derived EVs activated microglia to release large amounts of inflammatory cytokines, while treatment of microglia with mature neuron-derived EVs mitigated these effects. Our data suggest that NPC- and developing neuron-derived EVs are heterogenous vesicles loaded with differential cargo molecules, thus exerting a distinct capacity for the modulation of microglial activation (i.e., stimulation or restraint). Additionally, our experiments further support the proposal that communication between NPCs or developing neurons and microglia via EVs might play vital roles in regulating the transition of microglia from an activated to inactivated state.

Emerging studies suggest robust sex differences in microglial developmental trajectories and function in developing brain [71,72]. In an analysis of sex, age, and regional differences in microglial morphology in rats, Schwarz et al. [73] found that there are no sex differences in microglial number and morphology at embryonic day (E) 17. By postnatal (P) 4, sex has a significant effect on microglial phenotype. Males have more microglia of an activated morphology than females within the amygdala, parietal cortex, and hippocampus. These results indicate that sex differences in microglial development are associated with age and brain regions. Although we cultured mixed-sex microglia derived from VM of P1 rats, the sexual effect on EVs-elicited microglial activation might be not significant in our culture system based on above observations. Nevertheless, further research focusing on the role of sex in EVs-induced microglial activation is needed to more extensively investigate. Using in vitro culture of microglia derived from other postnatal brain regions, such as cortex, and hippocampus of male and female rats or injection of EVs into neonatal brains of male and female rats will make it more convincing.

It is well-known that the activation status of microglia and their expression of proinflammatory cytokines are closely related [45,46]. NF-κB and MAPKs play critical roles in the modulation of proinflammatory cytokine production. IκB masks NF-κB in the cytosol to prevent NF-κB-associated transcription in the nucleus. Stimulus-induced IκB degradation is triggered via phosphorylation by IKKs. Subsequently, phosphorylated IκB is targeted for ubiquitination and proteasomal degradation, resulting in the activation of NF-κB [51]. In the present study, we observed that the activation of MAPKs and IκBα phosphorylation and degradation, as well as the nuclear translocation of p65, were more obviously elicited by NPC- and immature neuron-derived EVs in microglia. Furthermore, pharmacological inhibition of MAPK signaling or NF-κB activation markedly attenuated the EV-induced production of cytokines. In line with a previous study reporting that astrocyte EVs activate microglia via the NF-κB signaling pathway [57], our findings further indicated that the activation of the MAPK and NF-κB signaling axes is responsible for the observed NPC- and immature neuron-derived EV-mediated activation of microglia.

MiRNAs are constituents of EVs which can be transferred from donor to recipient cells, thus affecting the functioning of distant and neighboring cells. Besides their conventional functions in post-transcriptional gene modulation, miRNAs can also act as signal molecules for the activation of membrane receptors. TLR7/8 (belonging to the endosomal receptors) are known to bind GU- or AU-rich single-stranded RNA ligands. Some miRNAs can serve as ligands for TLR7/8, thus inducing innate immune responses [74,75,76]. Specifically, EV-associated miRNAs carrying TLR7/8 binding motifs have been demonstrated to function as TLR7/8 agonists [55,56,77]. A previous study has demonstrated that NSC EVs shuttle miR-let-7 to microglia and mediate the morphological changes and activation of microglia via targeting of TLR7 [40]. In addition, miR-138-enriched astrocyte EVs have been shown to activate the TLR7–NF-κB axis in microglia to induce the expression of the proinflammatory cytokines TNF-α and IL-6 [57]. Our findings revealed that the blockade of TLR7 with an antagonist inhibited cytokine secretion, suggesting that the NPC- and immature neuron-derived EVs might exert biological functions on microglia, at least in part, in a TLR7-dependent fashion. TLR7 activation triggers the myeloid differentiation primary response 88 (MyD88)-dependent pathway, which subsequently leads to activation of NF-κB and MAPKs [60,61]. In the present study, we found that the EV-induced IκBα phosphorylation and degradation, as well as MAPK activation, were dampened when inhibiting TLR7. These results suggest that the NPC- and immature neuron-derived EVs activate microglia is mediated, at least partially, via TLR7–NF-κB and TLR7–MAPK signaling pathways. Morton et al. [40] have reported that miRNAs known to regulate microglial morphology and function, such as Let-7, miR-9, miR-26, and miR-181, are highly expressed in mice NSC EVs. Among these miRNAs, let-7 has been shown to modulate microglial activation by binding to TLR7 [54,75]. Thus, we postulate that miRNAs might function as potential effectors in NPC- and immature neuron-derived EVs for the induction of microglial activation. Whether or not these kinds of miRNAs containing G/U-rich motifs are enriched in NPC- and immature neuron-derived EVs and are transferred to microglia remains a subject for future investigations.

Microglia are indispensable for neural development from neurogenesis to cell survival, as well as synapse formation and elimination. Considering the spatiotemporally dynamic changes in the gene expression and morphology of microglia during brain development, it seems likely that the fates of developing neurons and microglia are intertwined. Previous work has shown that activated microglia are directly responsible for regulating neuron production in the embryonic prenatal cerebral cortex, acting by phagocytosing neural precursor cells [78]. Moreover, injection of conditioned medium from activated microglia induced by NSC exosomes, but not control microglia, has been shown to decrease the number of dividing NSCs in the lateral ventricles of P0 mice [40]. Using a combination of in vivo and in vitro approaches, Shigemoto-Mogami and colleagues [79] have demonstrated that activated microglia promote neurogenesis through the release of cytokines. In addition, activated microglia-derived insulin-like growth factor-1 has been shown to be required for the survival of layer V cortical neurons during postnatal development [80]. Taken together, these findings strongly suggest that microglia in the developing brain may sense microenvironmental changes and secrete a certain combination of cytokines or trophic factors to modulate neurogenesis and neuronal survival. In this report, we found that the activation of microglia by NPC- or immature neuron-derived EVs improved the survival, rather than the differentiation, of developing DA neurons. Activated microglia-released proinflammatory cytokines (i.e., TNF-α, IL-1β, IL-6, and IFN-γ) were found to promote neurogenesis of neurospheres [79], whereas the protective effect of these cytokines on developing neurons remains unclear. Investigation of the roles of these cytokines in improving neuronal survival by application of antibodies into the microglia-conditioned medium requires further exploration. Notably, activated microglia also secrete a set of trophic factors, which may contribute to the survival of developing neurons [16]. Our results indicated that, in our culture system, NPC- or immature neuron-derived EVs exert significant effects on microglial signaling, forming a positive feedback loop affecting DA neuronal survival during the late development period.

## 4. Materials and Methods

### 4.1. Neural Progenitor Cell Cultures

Cultures of mixed-sex embryonic NPCs were performed as described previously [62]. Briefly, VM tissues were dissected from E13.5 Sprague Dawley (SD) rats and meninges and blood vessels were removed. VM tissues were dissociated enzymatically (TrypLE^TM^ Express, Invitrogen, Carlsbad, CA, USA) and mechanically triturated and plated into 60 mm dishes (8 × 10^5^ cells/dish) precoated with 15 μg/mL poly-L-ornithine (Sigma-Aldrich, St. Louis, MO, USA) and 20 μg/mL fibronectin (Invitrogen). Cells were cultured in serum-free NPC culture medium containing Dulbecco’s Modified Eagle’s Medium (DMEM)-Ham’s F12, 2% B27 supplement (Invitrogen), 0.6% glucose, 20 ng/mL bFGF (Sigma-Aldrich), 20 ng/mL EGF (Invitrogen), 2 μg/mL heparin (Sigma-Aldrich), 100 U/mL penicillin, and 100 μg/mL streptomycin. Cultures were replaced with half of the fresh NPC medium 3 days later and were used for isolation of EVs 7 days later.

### 4.2. Mesencephalic Neuron-Enriched Cultures

Mixed-sex embryonic mesencephalic neuron-enriched cultures derived from VM at E14.5 were prepared as described previously [81]. Briefly, dissected tissues were dissociated enzymatically and mechanically. Cells were resuspended in neurobasal medium containing 0.5 mM glutamine, 25 μM glutamate, and 2% B27 supplement and seeded into 35 mm culture dishes (4 × 10^6^ cells/dish) precoated with poly-D-lysine (20 μg/mL). Four days later, the medium was changed to fresh neurobasal/B27 medium without glutamate, and the medium was replaced every 3 days thereafter. Neuron-enriched cultures at different culture periods were used to isolate EVs.

### 4.3. Microglial Cultures

Mixed-sex postnatal microglia were prepared from the VM of 1-day-old SD rats as previously described [82]. Briefly, VM tissues, devoid of meninges and blood vessels, were dissociated by enzymatic digestion (0.2% trypsin) and mild mechanical trituration. The isolated cells were seeded into poly-D-lysine-coated T175 tissue culture flasks in DMEM containing 10% FBS, 100 U/mL penicillin, and 100 μg/mL streptomycin. The medium was changed 4 days later. Upon reaching confluence (12–14 days), microglia were isolated from the mixed glial cultures by shaking the flasks on an orbital shaker at 180 rpm for 2 h. Detached cells were plated into a 24-well plate at a density of 3 × 10^5^ cells per well. After 2 h of incubation at 37 °C, nonadherent cells were removed. The purity of microglia cultures was assessed by using Iba1 antibody, and more than 97% of cells were stained positively (Figure 2A). Cells were cultured for 2 days before treatment with NPC- or neuron-derived EVs (2 × 10^10^ particles/well). For collection of conditioned media, microglia were treated with EVs for 24 h. The supernatants were collected and kept frozen at –80 °C until use.

### 4.4. Dopaminergic Neuron Differentiation Assay

Mixed-sex embryonic VM precursor cells were prepared using a previously described protocol [62]. VM tissues were dissected from E14.5 SD rats, then dissociated enzymatically and mechanically. Cells were seeded into 24-well (4 × 10^5^ cells/well) culture plates precoated with poly-D-lysine, then maintained in 0.5 mL/well of minimum essential medium (MEM) supplemented with 10% FBS, 10% horse serum, 1 g/L glucose, 2 mM L-glutamine, 1 mM sodium pyruvate, 100 μM non-essential amino acids, 100 U/mL penicillin, and 100 μg/mL streptomycin. For DA neuron differentiation, at 4 h after seeding, cultures were switched to DMEM/F12/N2 medium in the presence of 35% conditioned medium from control or EV-stimulated microglia for the indicated periods.

### 4.5. Isolation of EVs

EVs were prepared from conditioned media derived from NPCs (DIV7) and mesencephalic neuron-enriched cultures (DIV7-15) by ultracentrifugation as previously described [83]. In brief, the culture media were collected and spun at 300× *g* for 10 min to remove cell debris. The supernatants were then sequentially centrifuged at 10,000× *g* for 40 min and 100,000× *g* for 90 min at 4 °C. The pellets containing EVs were washed once with cold PBS and centrifuged again at 100,000× *g* for 90 min. Unused EVs were stored at −80 °C as PBS suspensions. Protein concentrations in the EV preparations were quantified by Bradford assay (Bio-Rad, Hercules, CA, USA). The size distribution and nanoparticle concentration were evaluated by nanoparticle tracking analysis. The common markers of EVs were detected by Western blotting.

### 4.6. Quantification and Size Evaluation of EVs

To evaluate the size distribution and quantity of the EVs in more detail, the NanoSight NS300 nanoparticle characterization system (Malvern Panalytical, Malvern, UK) equipped with Green laser (532 nm) illumination was used for real-time characterization of the EV preparations. All samples were appropriately diluted in saline solution to a final volume of 1 mL. The ideal measurement concentrations were found through pre-testing to determine the optimal particle per frame value (20–100 particles/frame). The capture settings were set according to the manufacturer’s software manual (NanoSight NS300 User Manual). To calculate the mean size and particle counts, the software NTA 3.4 was used for analysis of three 60 s videos per sample with a detection threshold of 5.

### 4.7. Labeling and Uptake of EVs

EVs were fluorescence-labeled with PKH67 (Sigma-Aldrich), a lipophilic dye, according to the manufacturer’s protocol. EVs were suspended in diluent C (1 mL), then incubated with PKH67 solution (1 mL) for 5 min at room temperature. As negative control, PBS was also incubated with PKH67. 2 mL 1% bovine serum albumin (BSA) was added to stop labeling. Then, the mixture was ultracentrifuged (100,000× *g*) for 90 min to obtain EVs precipitate, followed by washing again with PBS (100,000× *g*) for 90 min. Finally, PKH67-labeled EVs were resuspended in PBS. Subsequently, the PKH67-labeled EVs or PBS were incubated with microglia for 1 h. After the cells were fixed, we stained the cells with Iba1 antibody and DAPI. The images were obtained under a confocal microscope.

### 4.8. Real-Time RT-PCR Analysis

Total RNA was extracted from EV-treated microglia with TRIzol^®^ reagent (Invitrogen). After synthesis of cDNA from total RNA using SuperScript^TM^ III Reverse transcriptase (Invitrogen), SYBR Green chemistry in conjunction with real-time PCR was used to determine the expression of genes (Power SYBR^®^ Green PCR Master Mix, Applied Biosystems, Foster City, CA, USA). The primer sequences were as follows: *TNF-α*, 5′-CAG GGC AAT GAT CCC AAA GTA-3′ and 5′-GCA GTC AGA TCA TCT TCT CGA-3′; *IL-1β*, 5′-AGG CTT CCT TGT GCA AGT GT-3′ and 5′-TGA GTG ACA CTG CCT TCC TG-3′; *IL-6*, 5′-CCG GAG AGG AGA CTT CAC AG-3′ and 5′-CAG AAT TGC CAT TGC ACA AC-3′; *β-actin*, 5′-CAC CCG CGA GTA CAA CCT TC-3′ and 5′-CCC ATA CCC ACC ATC ACA CC-3′. The threshold cycle (C_t_) value for each test gene was normalized to the C_t_ value for the β-actin control from the same RNA preparation. The ratio of transcription of each gene was calculated as 2^−(△Ct)^, where ΔC_t_ denotes the difference C_t(test gene)_ − C_t(β-actin)_.

### 4.9. Western Blotting

Cells or EVs were lysed in M-PER^®^ mammalian protein extraction reagent (Thermo Scientific, Waltham, MA, USA) containing 5 mM sodium orthovanadate and protease inhibitor cocktail (Roche, Mannheim, Germany). The protein concentration in samples was determined by Bradford assay (Bio-Rad). Proteins were separated on 10~12% sodium dodecyl sulfate-polyacrylamide gel (SDS-PAGE) and transferred to immobilon polyvinylidene difluoride (PVDF) membranes (Merck Millipore, Billerica, MA, USA). The membranes were incubated in Tris-buffered saline Tween 20 (TBST, 0.1 M Tris/HCl, pH 7.4, 0.9% NaCl, 0.1% Tween 20) consisting of 5% skimmed milk powder for 1 h to block non-specific binding. After rinsing with TBST buffer, the membranes were incubated with the primary antibodies diluted in blocking solution overnight at 4 °C. The antibodies used were as follows: mouse anti-Alix, mouse anti-flotillin-1 (both from BD Biosciences, Franklin Lakes, NJ, USA), rabbit anti-tsg101 (Abcam, Cambridge, MA, USA), rabbit anti-calnexin (Millipore), rabbit anti-synapsin-1, rabbit anti-synaptophysin, rabbit anti-α-synuclein, rabbit anti-PSD95, rabbit anti-CD9, rabbit anti-phospho-IκBα (Ser32), rabbit anti-IκBα, rabbit anti-phospho-NF-κB p65 (Ser468, Ser536), rabbit anti-NF-κB p65, rabbit anti-phospho-p38, rabbit anti-p38, rabbit anti-phospho-ERK, rabbit anti-ERK, rabbit anti-phospho-JNK, and rabbit anti-JNK (all from Cell Signaling Technology, Beverly, MA, USA). Antibody against β-actin was used as an internal control to determine loading efficiency. The membranes were washed three times with TBST followed by incubation with appropriate horseradish peroxidase-conjugated secondary antibodies. Antigen–antibody complexes were detected using an ECL chemiluminescence detection system (PerkinElmer, Boston, MA, USA). The intensity of the bands was quantified with a GS-900™ calibrated densitometer (Bio-Rad) and calculated as the optical density × area of bands using the Image Lab 6.0 software. The band intensities of phosphorylated proteins were normalized to their total proteins. Considering the degradation of IκBα occurs upon IκBα phosphorylation, the band intensities of both phosphorylated and total IκBα proteins were normalized to β-actin. This normalized value was displayed as a relative densitometric bar graph.

### 4.10. Immunocytochemistry

Microglia identification and the translocation of NF-κB p65 from cytosol to the nucleus were detected by immunostaining with anti-Iba1 antibody (Wako, Chuo-Ku, Osaka, Japan) and anti-p65 antibody, respectively. Microglial cultures were fixed with 4% paraformaldehyde, followed by blocking with PBS containing 0.3% Triton X-100 and 2% BSA for 1 h at room temperature. After blocking, cells were incubated overnight at room temperature with anti-Iba1 antibody or anti-p65 antibody. For fluorescence labeling experiments, cells were incubated for 1 h at room temperature with the secondary antibody conjugated to the Alexa Fluor-488 or -647 (Jackson Immuno Research, West Grove, PA, USA). Samples were counterstained with DAPI (1 μg/mL, Sigma-Aldrich) and mounted with 50% glycerol in PBS. Microscopic observations were performed with a Zeiss LSM 510 META confocal imaging system (Carl Zeiss, Oberkochen, Germany). The fluorescence intensity in the nucleus was analyzed using the ImageJ 1.54p software. For the detection of DA neurons, VM precursor cells were fixed, permeabilized, and incubated with rabbit anti-tyrosine hydroxylase (TH) antibody (Millipore). After washing, the bound anti-TH antibody was visualized by incubation with an appropriate biotinylated secondary antibody followed by the Vectastain avidin-biotin-peroxidase (ABC) reagents (Vector Laboratories, Burlingame, CA, USA) and color development with 3,3′-diaminobenzidine. The number of TH-positive neurons was counted throughout the entire surface area of each culture well.

### 4.11. Cytokines Assay

Microglia were stimulated with EVs, and supernatants were collected and kept frozen in aliquots at –80 °C until use. The release of TNF-α, IL-1β, and IL-6 was measured with a commercial enzyme-linked immunosorbent assay (ELISA) kit from R&D Systems (Minneapolis, MN, USA), according to the manufacturer’s protocol.

### 4.12. Statistical Analysis

All data are expressed as mean ± SEM. Data were analyzed by one-way ANOVA followed by Scheffe’s test. For paired analyses, a *t*-test was performed. A *p*-value less than 0.05 was considered statistically significant.

## 5. Conclusions

The findings of this study demonstrated that NPCs and developing neurons might play crucial roles in the induction or restriction of microglial activation through the release of EVs. NPC- and immature neuron-derived EVs were found to evoke microglial activation via the TLR7-dependent activation of MAPK and NF-κB signaling pathways. Furthermore, microglial activation caused by NPC- or immature neuron-derived EVs showed a consequent beneficial effect regarding the survival of developing DA neurons. During neuronal development, it is conceivable and likely that neuronal EV compositions, such as miRNAs, change over time and thus discrepantly affect the activities and functions of microglia. Nevertheless, the contribution of NPC- and developing neuron-derived EVs to the modulation of the developmental phase transformation of microglia in vivo needs to be validated. To address this issue, the expression of inflammatory cytokine genes in microglia isolated from brains [57] and the observation of Iba1-positive microglial morphology (i.e., amoeboid or ramified phenotype) [20] after the injection of NPC and developing neuron-derived EVs into neonatal brains will be determined in the future.

## Data Availability

Data is contained within the article.

## References

[1] Kierdorf K., Prinz M. (2017). Microglia in steady state. J. Clin. Investig..

[2] Mosser C.A., Baptista S., Arnoux I., Audinat E. (2017). Microglia in CNS development: Shaping the brain for the future. Prog. Neurobiol..

[3] Lenz K.M., Nelson L.H. (2018). Microglia and beyond: Innate immune cells as regulators of brain development and behavioral function. Front. Immunol..

[4] Li Q., Barres B.A. (2018). Microglia and macrophages in brain homeostasis and disease. Nat. Rev. Immunol..

[5] Salter M.W., Stevens B. (2017). Microglia emerge as central players in brain disease. Nat. Med..

[6] Alliot F., Godin I., Pessac B. (1999). Microglia derive from progenitors, originating from the yolk sac, and which proliferate in the brain. Brain Res. Dev. Brain Res..

[7] Chan W.Y., Kohsaka S., Rezaie P. (2007). The origin and cell lineage of microglia: New concepts. Brain Res. Rev..

[8] Ginhoux F., Greter M., Leboeuf M., Nandi S., See P., Gokhan S., Mehler M.F., Conway S.J., Ng L.G., Stanley E.R. (2010). Fate mapping analysis reveals that adult microglia derive from primitive macrophages. Science.

[9] Kierdorf K., Erny D., Goldmann T., Sander V., Schulz C., Perdiguero E.G., Wieghofer P., Heinrich A., Riemke P., Hölscher C. (2013). Microglia emerge from erythromyeloid precursors via Pu.1- and Irf8-dependent pathways. Nat. Neurosci..

[10] Ginhoux F., Garel S. (2018). The mysterious origins of microglia. Nat. Neurosci..

[11] Navascués J., Calvente R., Marín-Teva J.L., Cuadros M.A. (2000). Entry, dispersion and differentiation of microglia in the developing central nervous system. An. Acad. Bras. Cienc..

[12] Rigato C., Buckinx R., Le-Corronc H., Rigo J.M., Legendre P. (2011). Pattern of invasion of the embryonic mouse spinal cord by microglial cells at the time of the onset of functional neuronal networks. Glia.

[13] Swinnen N., Smolders S., Avila A., Notelaers K., Paesen R., Ameloot M., Brône B., Legendre P., Rigo J.M. (2013). Complex invasion pattern of the cerebral cortex by microglial cells during development of the mouse embryo. Glia.

[14] Bruttger J., Karram K., Wörtge S., Regen T., Marini F., Hoppmann N., Klein M., Blank T., Yona S., Wolf Y. (2015). Genetic cell ablation reveals clusters of local self-renewing microglia in the mammalian central nervous system. Immunity.

[15] Hattori Y. (2022). The behavior and functions of embryonic microglia. Anat. Sci. Int..

[16] Nayak D., Roth T.L., McGavern D.B. (2014). Microglia development and function. Annu. Rev. Immunol..

[17] Hattori Y. (2023). The multifaceted roles of embryonic microglia in the developing brain. Front. Cell. Neurosci..

[18] Matcovitch-Natan O., Winter D.R., Giladi A., Vargas Aguilar S., Spinrad A., Sarrazin S., Ben-Yehuda H., David E., Zelada González F., Perrin P. (2016). Microglia development follows a stepwise program to regulate brain homeostasis. Science.

[19] Hattori Y., Itoh H., Tsugawa Y., Nishida Y., Kurata K., Uemura A., Miyata T. (2022). Embryonic pericytes promote microglial homeostasis and their effects on neural progenitors in the developing cerebral cortex. J. Neurosci..

[20] Scott E.P., Breyak E., Nishinakamura R., Nakagawa Y. (2022). The zinc finger transcription factor Sall1 is required for the early developmental transition of microglia in mouse embryos. Glia.

[21] Biber K., Neumann H., Inoue K., Boddeke H.W. (2007). Neuronal ‘On’ and ‘Off’ signals control microglia. Trends Neurosci..

[22] Hoek R.M., Ruuls S.R., Murphy C.A., Wright G.J., Goddard R., Zurawski S.M., Blom B., Homola M.E., Streit W.J., Brown M.H. (2000). Down-regulation of the macrophage lineage through interaction with OX2 (CD200). Science.

[23] Cardona A.E., Pioro E.P., Sasse M.E., Kostenko V., Cardona S.M., Dijkstra I.M., Huang D., Kidd G., Dombrowski S., Dutta R. (2006). Control of microglial neurotoxicity by the fractalkine receptor. Nat. Neurosci..

[24] Basso M., Bonetto V. (2016). Extracellular vesicles and a novel form of communication in the brain. Front. Neurosci..

[25] Lai C.P., Breakefield X.O. (2012). Role of exosomes/microvesicles in the nervous system and use in emerging therapies. Front. Physiol..

[26] Pegtel D.M., Peferoen L., Amor S. (2014). Extracellular vesicles as modulators of cell-to-cell communication in the healthy and diseased brain. Philos. Trans. R. Soc. Lond. Ser. B. Biol. Sci..

[27] Théry C., Witwer K.W., Aikawa E., Alcaraz M.J., Anderson J.D., Andriantsitohaina R., Antoniou A., Arab T., Archer F., Atkin-Smith G.K. (2018). Minimal information for studies of extracellular vesicles 2018 (MISEV2018): A position statement of the International Society for Extracellular Vesicles and update of the MISEV2014 guidelines. J. Extracell. Vesicles.

[28] Mause S.F., Weber C. (2010). Microparticles: Protagonists of a novel communication network for intercellular information exchange. Circ. Res..

[29] Raposo G., Stoorvogel W. (2013). Extracellular vesicles: Exosomes, microvesicles, and friends. J. Cell Biol..

[30] Frühbeis C., Fröhlich D., Kuo W.P., Krämer-Albers E.M. (2013). Extracellular vesicles as mediators of neuron-glia communication. Front. Cell. Neurosci..

[31] Pistono C., Bister N., Stanová I., Malm T. (2021). Glia-derived extracellular vesicles: Role in central nervous system communication in health and disease. Front. Cell Dev. Biol..

[32] Schnatz A., Müller C., Brahmer A., Krämer-Albers E.M. (2021). Extracellular vesicles in neural cell interaction and CNS homeostasis. FASEB Bioadv..

[33] Bahrini I., Song J.H., Diez D., Hanayama R. (2015). Neuronal exosomes facilitate synaptic pruning by up-regulating complement factors in microglia. Sci. Rep..

[34] Jiang D., Gong F., Ge X., Lv C., Huang C., Feng S., Zhou Z., Rong Y., Wang J., Ji C. (2020). Neuron-derived exosomes-transmitted miR-124-3p protect traumatically injured spinal cord by suppressing the activation of neurotoxic microglia and astrocytes. J. Nanobiotechnol..

[35] Peng H., Harvey B.T., Richards C.I., Nixon K. (2021). Neuron-derived extracellular vesicles modulate microglia activation and function. Biology.

[36] Xian X., Cai L.L., Li Y., Wang R.C., Xu Y.H., Chen Y.J., Xie Y.H., Zhu X.L., Li Y.F. (2022). Neuron secrete exosomes containing miR-9-5p to promote polarization of M1 microglia in depression. J. Nanobiotechnol..

[37] Thion M.S., Garel S. (2017). On place and time: Microglia in embryonic and perinatal brain development. Curr. Opin. Neurobiol..

[38] Feliciano D.M., Zhang S., Nasrallah C.M., Lisgo S.N., Bordey A. (2014). Embryonic cerebrospinal fluid nanovesicles carry evolutionarily conserved molecules and promote neural stem cell amplification. PLoS ONE.

[39] Morton M.C., Feliciano D.M. (2016). Neurovesicles in brain development. Cell. Mol. Neurobiol..

[40] Morton M.C., Neckles V.N., Seluzicki C.M., Holmberg J.C., Feliciano D.M. (2018). Neonatal subventricular zone neural stem cells release extracellular vesicles that act as a microglial morphogen. Cell Rep..

[41] Ma Y., Li C., Huang Y., Wang Y., Xia X., Zheng J.C. (2019). Exosomes released from neural progenitor cells and induced neural progenitor cells regulate neurogenesis through miR-21a. Cell Commun. Signal..

[42] Stronati E., Conti R., Cacci E., Cardarelli S., Biagioni S., Poiana G. (2019). Extracellular vesicle-induced differentiation of neural stem progenitor cells. Int. J. Mol. Sci..

[43] Andreeva N., Heldt J., Leclere N., Gross J. (2001). Differential response of immature and mature neurons to hypoxia in rat mesencephalic cultures. Dev. Neurosci..

[44] Lesuisse C., Martin L.J. (2002). Long-term culture of mouse cortical neurons as a model for neuronal development, aging, and death. J. Neurobiol..

[45] Fernández-Arjona M.D.M., Grondona J.M., Granados-Durán P., Fernández-Llebrez P., López-Ávalos M.D. (2017). Microglia morphological categorization in a rat model of neuroinflammation by hierarchical cluster and principal components analysis. Front. Cell. Neurosci..

[46] Fernández-Arjona M.D.M., Grondona J.M., Fernández-Llebrez P., López-Ávalos M.D. (2019). Microglial morphometric parameters correlate with the expression level of IL-1beta, and allow identifying different activated morphotypes. Front. Cell. Neurosci..

[47] Koistinaho M., Koistinaho J. (2002). Role of p38 and p44/42 mitogen-activated protein kinases in microglia. Glia.

[48] Kaminska B., Gozdz A., Zawadzka M., Ellert-Miklaszewska A., Lipko M. (2009). MAPK signal transduction underlying brain inflammation and gliosis as therapeutic target. Anat. Rec..

[49] Hayden M.S., Ghosh S. (2008). Shared principles in NF-κB signaling. Cell.

[50] Oeckinghaus A., Hayden M.S., Ghosh S. (2011). Crosstalk in NF-κB signaling pathways. Nat. Immunol..

[51] Karin M., Ben-Neriah Y. (2000). Phosphorylation meets ubiquitination: The control of NF-κB activity. Annu. Rev. Immunol..

[52] Forsbach A., Nemorin J.G., Montino C., Müller C., Samulowitz U., Vicari A.P., Jurk M., Mutwiri G.K., Krieg A.M., Lipford G.B. (2008). Identification of RNA sequence motifs stimulating sequence-specific TLR8-dependent immune responses. J. Immunol..

[53] Fabbri M., Paone A., Calore F., Galli R., Gaudio E., Santhanam R., Lovat F., Fadda P., Mao C., Nuovo G.J. (2012). MicroRNAs bind to Toll-like receptors to induce prometastatic inflammatory response. Proc. Natl. Acad. Sci. USA.

[54] Lehmann S.M., Krüger C., Park B., Derkow K., Rosenberger K., Baumgart J., Trimbuch T., Eom G., Hinz M., Kaul D. (2012). An unconventional role for miRNA: Let-7 activates Toll-like receptor 7 and causes neurodegeneration. Nat. Neurosci..

[55] Hu G., Liao K., Niu F., Yang L., Dallon B.W., Callen S., Tian C., Shu J., Cui J., Sun Z. (2018). Astrocyte EV-induced lincRNA-Cox2 regulates microglial phagocytosis: Implications for morphine-mediated neurodegeneration. Mol. Ther. Nucleic Acids.

[56] Xu J., Feng Y., Jeyaram A., Jay S.M., Zou L., Chao W. (2018). Circulating plasma extracellular vesicles from septic mice induce inflammation via microRNA- and TLR7-dependent mechanisms. J. Immunol..

[57] Liao K., Niu F., Hu G., Yang L., Dallon B., Villarreal D., Buch S. (2020). Morphine-mediated release of miR-138 in astrocyte-derived extracellular vesicles promotes microglial activation. J. Extracell. Vesicles.

[58] Schrezenmeier E., Dörner T. (2020). Mechanisms of action of hydroxychloroquine and chloroquine: Implications for rheumatology. Nat. Rev. Rheumatol..

[59] Kuznik A., Bencina M., Svajger U., Jeras M., Rozman B., Jerala R. (2011). Mechanism of endosomal TLR inhibition by antimalarial drugs: Implications for drug design. J. Immunol..

[60] Duan T., Du Y., Xing C., Wang H.Y., Wang R.F. (2022). Toll-like receptor signaling and its role in cell-mediated immunity. Front. Immunol..

[61] Zheng H., Wu P., Bonnet P.A. (2023). Recent advances on small-molecule antagonists targeting TLR7. Molecules.

[62] Huang H.Y., Chiu T.L., Chang H.F., Hsu H.R., Pang C.Y., Liew H.K., Wang M.J. (2015). Epigenetic regulation contributes to urocortin-enhanced midbrain dopaminergic neuron differentiation. Stem Cells.

[63] Zusso M., Methot L., Lo R., Greenhalgh A.D., David S., Stifani S. (2012). Regulation of postnatal forebrain amoeboid microglial cell proliferation and development by the transcription factor Runx1. J. Neurosci..

[64] Schilling T., Nitsch R., Heinemann U., Haas D., Eder C. (2001). Astrocyte-released cytokines induce ramification and outward K+ channel expression in microglia via distinct signaling pathways. Eur. J. Neurosci..

[65] Wollmer M.A., Lucius R., Wilms H., Held-Feindt J., Sievers J., Mentlein R. (2001). ATP and adenosine induce ramification of microglia in vitro. J. Neuroimmunol..

[66] McKinsey G.L., Santander N., Zhang X., Kleemann K.L., Tran L., Katewa A., Conant K., Barraza M., Waddell K., Lizama C.O. (2025). Radial glia integrin avb8 regulates cell autonomous microglial TGFbeta1 signaling that is necessary for microglial identity. Nat. Commun..

[67] Mosher K.I., Andres R.H., Fukuhara T., Bieri G., Hasegawa-Moriyama M., He Y., Guzman R., Wyss-Coray T. (2012). Neural progenitor cells regulate microglia functions and activity. Nat. Neurosci..

[68] Arnò B., Grassivaro F., Rossi C., Bergamaschi A., Castiglioni V., Furlan R., Greter M., Favaro R., Comi G., Becher B. (2014). Neural progenitor cells orchestrate microglia migration and positioning into the developing cortex. Nat. Commun..

[69] Wu H.M., Zhang L.F., Ding P.S., Liu Y.J., Wu X., Zhou J.N. (2014). Microglial activation mediates host neuronal survival induced by neural stem cells. J. Cell Mol. Med..

[70] de Almeida M.M.A., Goodkey K., Voronova A. (2023). Regulation of microglia function by neural stem cells. Front. Cell. Neurosci..

[71] Bordt E.A., Ceasrine A.M., Bilbo S.D. (2020). Microglia and sexual differentiation of the developing brain: A focus on ontogeny and intrinsic factors. Glia.

[72] VanRyzin J.W., Marquardt A.E., Pickett L.A., McCarthy M.M. (2020). Microglia and sexual differentiation of the developing brain: A focus on extrinsic factors. Glia.

[73] Schwarz J.M., Sholar P.W., Bilbo S.D. (2012). Sex differences in microglial colonization of the developing rat brain. J. Neurochem..

[74] Feng Y., Zou L., Yan D., Chen H., Xu G., Jian W., Cui P., Chao W. (2017). Extracellular microRNAs induce potent innate immune responses via TLR7/MyD88-dependent mechanisms. J. Immunol..

[75] Buonfiglioli A., Efe I.E., Guneykaya D., Ivanov A., Huang Y., Orlowski E., Krüger C., Deisz R.A., Markovic D., Flüh C. (2019). let-7 microRNAs regulate microglial function and suppress glioma growth through Toll-like receptor 7. Cell Rep..

[76] Wu N., Morsey B.M., Emanuel K.M., Fox H.S. (2021). Sequence-specific extracellular microRNAs activate TLR7 and induce cytokine secretion and leukocyte migration. Mol. Cell. Biochem..

[77] Donzelli J., Proestler E., Riedel A., Nevermann S., Hertel B., Guenther A., Gattenlöhner S., Savai R., Larsson K., Saul M.J. (2021). Small extracellular vesicle-derived miR-574-5p regulates PGE2-biosynthesis via TLR7/8 in lung cancer. J. Extracell. Vesicles.

[78] Cunningham C.L., Martínez-Cerdeño V., Noctor S.C. (2013). Microglia regulate the number of neural precursor cells in the developing cerebral cortex. J. Neurosci..

[79] Shigemoto-Mogami Y., Hoshikawa K., Goldman J.E., Sekino Y., Sato K. (2014). Microglia enhance neurogenesis and oligodendrogenesis in the early postnatal subventricular zone. J. Neurosci..

[80] Ueno M., Fujita Y., Tanaka T., Nakamura Y., Kikuta J., Ishii M., Yamashita T. (2013). Layer V cortical neurons require microglial support for survival during postnatal development. Nat. Neurosci..

[81] Huang H.Y., Lin S.Z., Kuo J.S., Chen W.F., Wang M.J. (2007). G-CSF protects dopaminergic neurons from 6-OHDA-induced toxicity via the ERK pathway. Neurobiol. Aging.

[82] Wang M.J., Lin S.Z., Kuo J.S., Huang H.Y., Tzeng S.F., Liao C.H., Chen D.C., Chen W.F. (2007). Urocortin modulates inflammatory response and neurotoxicity induced by microglial activation. J. Immunol..

[83] Jovičić A., Gitler A.D. (2017). Distinct repertoires of microRNAs present in mouse astrocytes compared to astrocyte-secreted exosomes. PLoS ONE.

